# Role of Arginine 117 in Substrate Recognition by Human Cytochrome P450 2J2

**DOI:** 10.3390/ijms19072066

**Published:** 2018-07-16

**Authors:** Pierre Lafite, François André, Joan P. Graves, Darryl C. Zeldin, Patrick M. Dansette, Daniel Mansuy

**Affiliations:** 1 Laboratoire de Chimie et Biochimie Pharmacologiques et Toxicologiques, CNRS UMR8601, Université Paris Descartes, 75270 Paris CEDEX 06, France; pierre.lafite@univ-orleans.fr (P.L.); daniel.mansuy@parisdescartes.fr (D.M.); 2 Institute for Integrative Biology of the Cell (I2BC), DRF/Joliot/SB2SM, CEA, CNRS, Université Paris-Saclay, F-91198 Gif-sur-Yvette CEDEX, France; francois.andre@cea.fr; 3 Division of Intramural Research, National Institute of Environmental Health Sciences, Research Triangle Park, Durham, NC 27709, USA; graves@mail.nih.gov (J.P.G.); zeldin@niehs.nih.gov (D.C.Z.)

**Keywords:** human cytochrome P450, CYP2J2, mutants, regioselectivity, inhibitor, homology modeling, docking

## Abstract

The influence of Arginine 117 of human cytochrome P450 2J2 in the recognition of ebastine and a series of terfenadone derivatives was studied by site-directed mutagenesis. R117K, R117E, and R117L mutants were produced, and the behavior of these mutants in the hydroxylation of ebastine and terfenadone derivatives was compared to that of wild-type CYP2J2. The data clearly showed the importance of the formation of a hydrogen bond between R117 and the keto group of these substrates. The data were interpreted on the basis of 3D homology models of the mutants and of dynamic docking of the substrates in their active site. These modeling studies also suggested the existence of a R117-E222 salt bridge between helices B’ and F that would be important for maintaining the overall folding of CYP2J2.

## 1. Introduction

Cytochromes P450 (CYPs) form a huge superfamily of hemoproteins that play key roles in the metabolism of a large variety of xenobiotics and endogenous compounds [1]. In the human genome, 57 genes coding for CYPs have been identified [2]. CYP2J2 is the only member of the CYP2J subfamily in humans and is the only P450 that is mainly expressed in the cardiovascular system [3]. It is assumed to be the main arachidonic acid (AA) epoxygenase in the heart as the regio- and stereoselectivities of *cis*-epoxyeicosatrienoic acid (EET) formation by CYP2J2 match those in the human heart [3]. CYP2J2 is also expressed in the kidney, lung, and gastrointestinal tract, and to a lesser extent in the liver, pancreas, and brain [4,5,6,7,8,9]. EETs are important intracellular messengers in vascular tissues [7,8,9,10,11,12,13,14,15,16,17,18,19,20,21,22] and are also involved in processes related to cancer cell growth, angiogenesis, and tumor pathogenesis [7,8,9,21,22,23,24]. Several lines of evidence suggest that CYP2J2 promotes the neoplastic phenotype of carcinoma cells and stimulates metastasis [25,26], and therefore may represent a potential target for therapy of some human cancers [27].

**Figure 1:**
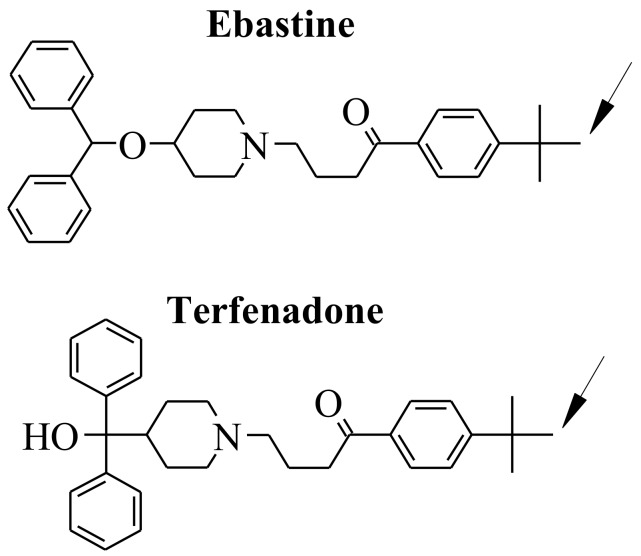
Structure of ebastine and terfenadone. The arrow represents the hydroxylation site by CYP2J2 [37,38,50].

CYP2J2 is involved in the oxidation of unsaturated fatty acids such as arachidonic, linoleic, docosahexaenoic, and eicosapentaenoic acids and some of their derivatives such as anandamide to biologically active epoxides [7,8,9,28,29,30,31,32,33,34,35]. It is also involved in the metabolism of drugs [7,8,9,36], such as ebastine [37,38,39] (Figure 1), astemizole [40], albendazone [36,41], amiodarone [36,42], and ritonavir [43]. Selective inhibitors of CYP2J2 have also been described [42,44,45,46,47,48,49]. Because of its roles in drug metabolism, in the physiology and pathophysiology of the cardiovascular system, and in cancer, it is important to better understand its active site structure and the mode of binding of its substrates and inhibitors. To date, no crystal structure of CYP2J2 has been published. However, several homology models have been reported for CYP2J2 and some of its mutants [30,36,50,51,52,53,54,55]. In that context, we have found that a series of terfenadone (Figure 1) analogs exhibited a very high affinity for CYP2J2 [44,45,50] with *K_M_* values up to 140 nM [50].

The regioselectivity of the CYP2J2-catalyzed oxidation of these analogs was surprising as it favored the less reactive homobenzylic position of the terminal alkyl chain. Docking of these substrates in a homology model of CYP2J2, that we have published in 2007, allowed us to interpret those results as these terfenadone derivatives appeared to bind in a hydrophobic channel whose extremity close to the heme only leads to restricted access of the substrate terminal alkyl chain to the iron [50]. In this model, the restricted access appeared to be due to a crown of bulky amino acid residues located just above the heme, and to the binding of the substrate CO group to Arg117 through a hydrogen bond. This hydrogen bond seems to be important for substrate recognition as replacement of the substrate CO group with a CH_2_ group led to a 10-fold decrease of affinity for CYP2J2 [45] and to a marked change of the hydroxylation regioselectivity [50]. Moreover, recent data about the binding of AA to CYP2J2 using homology modeling, induced fit docking, and molecular dynamics simulations were in favor of the binding of the AA carboxylate group to Arg117 [54].

To confirm the possible importance of Arg117 in substrate and inhibitor recognition by CYP2J2, we have produced several Arg117 mutants of CYP2J2 (CYP2J2-R117X), and compared the affinities of several terfenadone derivatives, bearing either the ketone function or a methylene function, towards CYP2J2 and its Arg117 mutants. We have also compared the regioselectivities of their hydroxylation by these proteins. Finally, construction of homology models of these mutants and dynamic docking of several substrates in their active sites allowed us to interpret the influence of R117 mutated residues on the recognition of terfenadone derivatives by the CYP2J2 mutants.

## 2. Results and Discussion

### 2.1. Expression and Stability of CYP2J2 R117 Mutants

**Figure 2:**
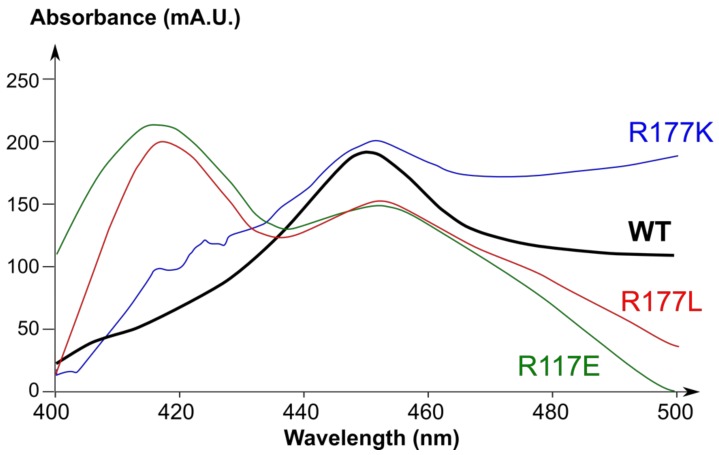
Fe(II)–CO difference visible spectra of recombinant wild-type and variant CYP2J2 proteins. All spectra were recorded at room temperature in 0.1 M phosphate buffer, pH 7.4.

Three CYP2J2 mutants in which the R117 residue was replaced with either a lysine, leucine, or glutamate residue were constructed. Wild-type CYP2J2 and its R117X mutants were coexpressed with human cytochrome P450 reductase in *Sf21* insect cells by using the baculovirus expression system. CYP2J2 expression levels ranged from 3 to 10 nmol P450 per liter of infected cells, depending on the mutant and the preparation. The expression levels and preparation-to-preparation variability were comparable with those obtained for other P450s by using a similar heterologous expression system [56,57]. Wild-type CYP2J2 and its R117K mutant exhibited typical cytochrome P450Fe(II)–CO difference spectra with a Soret peak at 450 nm (Figure 2). By contrast, the Fe(II)–CO difference spectra of the R117L and R117E variants exhibited a more intense peak at 420 nm (Figure 2). The latter difference spectra could be due to an improper protein folding and/or heme binding, as previously reported for other P450s [58,59]. In this regard, an ionic interaction between helices B’ and F, involving the R117 and E222 residues, has been described in a recent CYP2J2 homology model [51] and might be important for correct protein folding. Mutation of the basic R117 residue into a hydrophobic (R117L) or an acidic residue (R117E) might lead to the loss of this salt bridge between two structural elements of CYP2J2, whereas mutation to a positively charged lysine (R117K) might preserve this interaction, and thereby the tertiary structure of the protein.

### 2.2. Oxidation of Ebastine by CYP2J2 and Its R117 Mutants

Hydroxylation of ebastine by microsomes expressing CYP2J2 or its R117 mutants, in the presence of an NADPH-generating system, occurred in all cases at the level of the ebastine *t*-butyl moiety. The kinetic constants for the CYP2J2-catalyzed hydroxylation of ebastine (*K*_M_ of 0.3 µM and *k*_cat_/*K*_M_ of 133 min^−1^ µM^−1^) (Table 1) were in agreement with previously published values [50], whereas replacement of R117 with L117 led to a significant increase of the *K*_M_ for ebastine hydroxylation (Table 1). Interestingly, the R117E mutation led to a decrease of *K*_M_ (0.1 µM). The catalytic efficiency *k*_cat_/*K*_M_ found for CYP2J2 and its R117K and R117E mutants were in the same order of magnitude, whereas the *k*_cat_/*K*_M_ found for the R117L mutant was more than 10 times lower.

### 2.3. Inhibitory Effects of Terfenadone Derivatives on Ebastine Hydroxylation by CYP2J2 and Its R117 Mutants

In order to further analyze the role of arginine 117 in the binding of terfenadone derivatives, we studied the influence of R117 mutations on the inhibitory effects of a series of terfenadone derivatives, whose structures are shown in Table 2, on ebastine hydroxylation. All IC_50_ values were obtained from incubations with ebastine concentrations equal to the *K*_M_ values reported in Table 1, so that IC_50_ values should correspond to *K*_I_/2 where *K*_I_ is the inhibition constant of ebastine hydroxylation by each terfenadone derivative [60].

As previously reported [45], the compounds bearing a keto group (X = CO, Table 2), **1** and **2**, were the best inhibitors with IC_50_s IC_50_ values around 0.5 µM. In contrast, replacement of this CO group with a CH_2_ group, as in compounds **3** and **4**, led to a marked increase of the IC_50_ values (around 4 µM). We observed a very similar influence of the nature of the X group on the inhibitory effects of the terfenadone derivatives on ebastine hydroxylation by the R117K mutant, as the IC_50_s of compounds **1** and **2** were around 1 µM whereas those of compounds **3** and **4** were around 10 µM. The R117L mutant exhibited a different behavior as all the compounds bearing a CO or CH_2_ group led to IC_50_ values of around 2 µM. Finally, in the case of the R117E mutant, the IC_50_s of compounds **1** and **2** were 0.5 and 1 µM, respectively, whereas those of compounds bearing a CH_2_ group were around 2.5 µM.

### 2.4. Influence of R117 Mutations on the Regioselectivity of Terfenadone Derivatives Oxidation

As previously reported [50], the regioselectivity of the CYP2J2-catalyzed hydroxylation of terfenadone derivatives **2** and **5** for which X = CO was in favor of the β position (homobenzylic) of their alkyl chain **R**, even though their benzylic position (α) is the most chemically reactive one (Table 3), was observed. The regioselectivity of the CYP2J2-catalyzed hydroxylation of terfenadone derivative **4** in which X = CH_2_ was much less in favor of the homobenzylic position was also observed. Identical incubations of compounds **2**, **4**, and **5** with microsomes expressing the three R117 mutants led to hydroxylated metabolites identical to those previously obtained with microsomes expressing wild-type CYP2J2, as they exhibited identical HPLC retention times and MS spectra. The hydroxylation regioselectivities observed with the R117K mutant were very similar (compound **2**) if not identical (compounds **4** and **5**) to those found with wild-type CYP2J2. They were different in the case of the R117L mutant that led to a clear increase of the proportion of hydroxylation of the three substrates on the more chemically reactive benzylic (α) position (Table 3). Hydroxylation of compounds **2** and **5** by the R117E mutant occurred with regioselectivities almost identical to those found with wild-type CYP2J2; that found for compound **4** was more in favor of the homobenzylic position.

### 2.5. Homology Modeling of CYP2J2-R117X Mutants

Homology models of each CYP2J2-R117X mutant were constructed starting from the model we had previously built for CYP2J2 itself [50], in order to interpret the above results. After virtual mutagenesis of the R117 amino acid in the desired mutant, models were energetically minimized and thermally equilibrated at 300 K to take into account the diffusion of water molecules in the protein. Then, final models were obtained after an energy minimization step. All models were similar to our previously published model of CYP2J2 [50], and the global folding was identical to that found in published x-ray structures of mammalian P450s [61,62].

**Figure 3:**
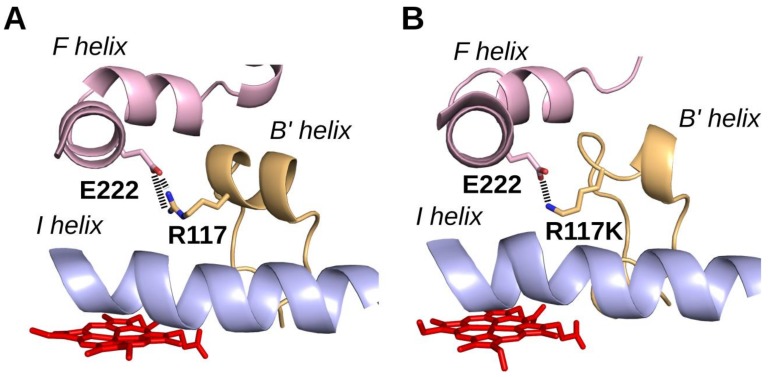
Interaction between B’ and F helices in CYP2J2 and its R117K mutant. (**A**): CYP2J2wt. (**B**): CYP2J2 R117K. The heme cofactor is rendered in red sticks, helices B’, F, and I are rendered as ribbons and colored in yellow, pink, and blue, respectively. The E222 and R/K117 amino acid side chains are represented as sticks, with oxygen and nitrogen atoms colored in red and dark blue, respectively. Putative salt bridges are represented by dashed lines.

Analysis of the interaction between the E222 and R117 amino acid residues of CYP2J2 showed the existence of a salt bridge between these two residues (Figure 3). A similar interaction was observed between E222 and K117 side chains in the case of the R117K mutant (Figure 3), which can be explained by the similar polarity and length of the arginine and lysine side chains. Replacement of R117 with a leucine or glutamate led to the loss of this salt bridge with the E222 residue (structures not shown). This E222-R117 salt bridge between B’ and F helices could be important for proper folding of the CYP2J2 protein. Li et al. [51] have also published a CYP2J2 homology model where R117 and E222 were linked by a salt bridge, and hypothesized that the R117 residue might be involved in a gating mechanism by opening and closing a cleft between B’ and F helices. The importance of the E222-R117 salt bridge for CYP2J2 protein stabilization would be in agreement with the spectral data of Figure 2. This stabilizing E222-R(or K)117 interaction would be present in CYP2J2 and its R117K mutant, in that both exhibited typical difference spectra of their Fe(II)–CO complexes characterized by a 450 nm peak. By contrast, the difference spectra of the Fe(II)–CO complexes of the R117L and R117E mutants that cannot establish such an interaction showed a peak at 420 nm and a much less intense peak at 450 nm, suggesting a less stable P450 form under the conditions used for recording the spectra.

### 2.6. Docking of Terfenadone Derivatives in the Active Site of CYP2J2 and Its Mutants

**Figure 4:**
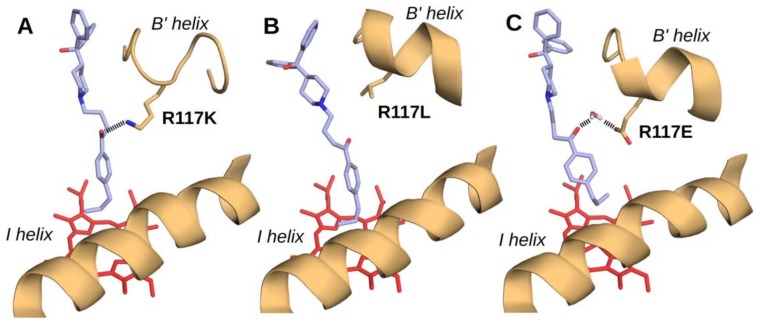
Docking of compound 2 in CYP2J2-R117X mutants active site model. (**A**) 0028 CYP2J2-R117K—compound **2**; (**B**) CYP2J2-R117L—compound **2**; (**C**) CYP2J2-R117E—compound **2** docking models. The representations of heme, helix B’, and R117X amino acid side chains are as in Figure 3. Water molecule and compound **2** are rendered as sticks in atom type color. Compound **2** carbon atoms are colored in light purple. Putative H-bonds are represented as dashed lines.

Docking of compounds **2** and **4** in the CYP2J2-R117X mutants active sites was performed using a previously described soft-restrained MD protocol [50]. In a general manner, docking of these two substrates in the three mutants led to a positioning similar to that previously found for terfenadone derivatives in the CYP2J2 active site [50] (Figure 4). Briefly, they were located in a hydrophobic cavity, their **R** terminal group pointing towards the heme and being surrounded by a crown of hydrophobic and bulky residues, namely I127, F310, A311, I375, I376, and V380 located just above the heme, that led to a restricted access to the iron [50]. The rest of the molecule was in a linear conformation, with the diphenyl moiety oriented towards the β-sheet domain. Figure 4A,B show that the hydrogen bond previously found between the CO group of compound **2** and arginine 117 in CYP2J2 [50] was replaced by an equivalent H-bond with lysine 117 in the R117K mutant complex with **2** (Figure 4A). In contrast, no such H-bond was present in the R117L mutant complex with **2** (Figure 4B). Figure 4C shows that glutamate 117 of the R117E mutant could establish a favorable H-bond interaction with the CO group of compound **2** (via an intermediate H_2_O molecule).

The distances between the heme iron atom and the carbon atoms of the **R** moiety of compound **2** calculated from these docked models are reported in Table 4. These distances and the binding modes found in Figure 4 were in agreement with the hydroxylation regioselectivities depicted in Table 3 and with the different inhibitor potencies of terfenadone derivatives towards ebastine hydroxylation by CYP2J2 and its mutants (Table 2). Actually, in the case of compounds in which X = CO (ebastine, **1**, **2**, and **5**), the existence of an H-bond between their CO group and R117 in CYP2J2, K117 in CYP2J2-R117K, or E117 (via an intermediate H_2_O molecule) in CYP2J2-R117E would explain at least in part the similar low *K_M_* values (0.1–0.4 µM) found for ebastine hydroxylation (Table 1) and the low IC_50_ values (0.4–1.6 µM) found for the inhibition of ebastine hydroxylation (Table 2). It would also explain in part the regioselectivities in favor of the less chemically reactive homobenzylic position observed in the hydroxylation of compounds **2** and **5** (87 to 97%) (Table 3).

For compounds **3** and **4**, X = CH_2_, which cannot establish such a favorable H-bond interaction, gave higher IC_50_ values for ebastine hydroxylation by CYP2J2, CYP2J2-R117K, and CYP2J2-R117E (2–13 µM; Table 2) than compounds **1** and **2** with X = CO. Moreover, the regioselectivities of the hydroxylation of compound **4** by CYP2J2, CYP2J2-R117K, and CYP2J2-R117E were less in favor of the homobenzylic position than those found in the case of compounds **2** and **5** (64–83% instead of 87–97%; Table 3).

In CYP2J2-R117L, no equivalent H-bond can exist with the substrate CO group, which would explain the increase of its *K_M_* value for ebastine hydroxylation (Table 1) and of the IC_50_ values of compounds **1** and **2** (Table 2), and the less regioselective hydroxylation of compounds **2** and **5** (Table 3), compared to the corresponding values for CYP2J2.

## 3. Conclusions

The aforementioned results on three site-directed mutants of CYP2J2 allowed us to elucidate the role of the R117 residue on the recognition of a series of terfenadone derivatives. Previous studies suggested that those compounds were bound in a hydrophobic pocket of CYP2J2 and pointed their terminal alkyl moiety towards the heme [50]. The access of this alkyl moiety to the heme iron was constrained by a crown of hydrophobic amino acid residues located above the heme and by the existence of an H-bond between their keto group and the arginine 117 residue. This constrained positioning would explain the surprising regioselectivity in favor of the homobenzylic position of the terminal alkyl chain of the terfenadone derivatives observed in their CYP2J2-catalyzed hydroxylation [50]. The above data on the three mutants R117X, with X = K, L, or E, showed that, in a general manner, these mutants also bind the various terfenadone derivatives used in this study and hydroxylate them on their terminal alkyl moiety. This indicates that the strong hydrophobic interactions occurring between those substrates and the active site amino acid residues play an important role in their binding. However, the importance of R117 in the binding of the terfenadone derivatives bearing a keto group (X = CO) was shown by the increase of the *K_M_* value of ebastine hydroxylation and the IC_50_ values of compounds **1** and **2**, and by the less regioselective hydroxylation of compound **2** upon mutation of R117 into L117. It is also shown by the increase of the IC_50_ values found for inhibition of CYP2J2 by compounds in which X = CH_2_ cannot establish an H-bond with R117, when compared to compounds for which X = CO. Accordingly, very similar IC_50_ values were found for the inhibition of CYP2J2-R117L-catalyzed ebastine hydroxylation by compounds bearing X = CO or CH_2_ groups. Finally, the R117K and R117E mutants that can also establish H-bonds with the compounds bearing a CO group (Figure 4A,C) behave in a manner similar to CYP2J2.

Besides its role in the recognition of ebastine and terfenadone derivatives by CYP2J2, R117 residue seems to also be involved in maintaining the tertiary structure of CYP2J2 by establishing an R117-E222 salt bridge between helices B’ and F (Figure 3). This is in agreement with the stability, under the conditions of Figure 2, of CYP2J2 and its R117K mutant in which a similar K117-E222 salt bridge can exist (Figure 3), and the relative instability of the R117L and R117E mutants in which such a salt bridge cannot exist. 

## 4. Materials and Methods

### 4.1. Materials

All chemicals used in this study were of highest purity available. Terfenadone and its derivatives **1**–**5** were synthesized as previously described [45].

### 4.2. Mutagenesis of the R117 Residue of the CYP2J2 cDNA

Three mutants of the CYP2J2 cDNA arginine residue at position 117 (R117) were generated in a previously described modified pAUw51-CYPOR baculovirus expression vector [63], using a QuikChange II XL Site-Directed Mutagenesis Kit (Stratagene, Les Ulis, France) following the manufacturer’s protocol. R117 (CGA) was mutated to a leucine residue (R117L with TTG), a lysine residue (R117K with AAA), or a glutamate residue (R117E with GAG). The following gene-specific mutagenic oligonucleotides were used:

5′-GGGAACCGGCCCGTGACCCCTATG**TTG**GAACATATCTTTAAGAAAAATGG-3′ and 5′-CCATTTTTCTTAAAGATATGTTC**CAA**CATAGGGGTCACGGGCCGGTTCCC-3′ for R117L;

5′-GGGAACCGGCCCGTGACCCCTATG**AAA**GAACATATCTTTAAGAAAAATGG-3′ and 5′-CCATTTTTCTTAAAGATATGTTC**TTT**CATAGGGGTCACGGGCCGGTTCCC-3′ for R117K; and 5′-GGGAACCGGCCCGTGACCCCTATG**GAG**GAACATATCTTTAAGAAAAATGG-3′ and 5′-CCATTTTTCTTAAAGATATGTTC**CTC**CATAGGGGTCACCCCCCGGTTCCC-3′ for R117E.

The mutations were confirmed by direct sequencing using ABI Big Dye reagent and an ABI 3100 sequencer (Applied Biosystems, Les Ulis, France).

### 4.3. Expression of the CYP2J2 R117 Mutants

Wild-type CYP2J2 and the three R117 mutants in a modified pAUw51-CYPOR baculovirus expression vector were cotransfected with Baculogold DNA (BD Biosciences, Pharmingen, Rungis, France) into *Spodopterafrugiperda Sf*21 insect cells and amplified. These amplified stocks were used to infect *Sf*21 insect cells for the collection of microsomal fractions as previously described [63]. Cytochrome P450 amounts were determined as previously described [64] and the enzyme concentration was measured as the P450 fraction of the mutants (A450–A490 from the Fe(II)–CO difference spectra). The difference spectra of the Fe(II)–CO complexes did not significantly change as a function of time.

### 4.4. Oxidation of Terfenadone Derivatives Assay and Analysis of Product Formation Regioselectivity

Identification and quantification of products formed upon CYP2J2-catalyzed oxidations were performed as reported previously [50]. Briefly, substrate (0.1–20 µM) and insect cells microsomes expressing CYP2J2 (1–5 nM P450) were preincubated at 37 °C in a shaking bath for 2–3 min in 0.1 M phosphate buffer, pH 7.4, containing 0.1 mM EDTA. Incubation was started (*t*_0_ = 0 min) with the addition of an NADPH-generating system (1 mM NADP^+^, 10 mM glucose 6-phosphate, and 2 units of glucose 6-phosphate dehydrogenase per mL) that was preincubated at 37 °C for 2 min. In the case of IC_50_ values determination, substrate (ebastine) concentration was set to the *K*_M_ value determined for each mutant. Usual incubation times were 2 to 5 min for kinetic constants and IC_50_ values determination and up to 30 min for metabolites identification. At *t_0_* and regularly thereafter, aliquots (200 µL) were taken and the reaction was terminated by treatment with 100 µL of a cold CH_3_CN/CH_3_COOH (10:1) mixture. Proteins were precipitated by centrifugation for 10 min at 10,000 rpm, and the supernatant was analyzed by reverse-phase HPLC. Protocol and apparatus used for kinetic constants determination using UV detection or for product identification using MS/MS^2^ were done as published previously [45,50]. The kinetic parameters correspond to initial rates that were measured under linear conditions for proteins and time.

### 4.5. Homology Modeling of CYP2J2-R117X Mutants and Docking of Derivatives 2 and 4 in CYP2J2-R117X Active Sites

The previously published 3D model of wild-type CYP2J2 [50] was used as a starting point for building the CYP2J2-R117X mutants models. R117 was mutated in silico using Pymol software (http://www.pymol.org) and the rotamer showing less steric clashes was chosen. The structural models of CYP2J2-R117X mutants were equilibrated by MD simulations of 1 ns in explicit solvent. All molecular dynamics simulations (MD) were carried out using the NAMD2 program [65], using the Amber force-field [66]. Topology and parameter files for heme-cysteinate were obtained from a previously published work [67]. Topology and parameter files for terfenadone derivatives were obtained using the antechamber program [68], using AM1-BCC charges [69]. The presence of water molecules in the active site was taken into account by solvation of the CYP2J2-R117X mutants in explicit solvent, using a periodic TIP3 water box with an edge set to 5 Å above the protein size. The whole system was then charge-neutralized by adding counter-ions and energy-minimized without restraints for 5000 steps using the steepest descent minimization to remove residual steric clashes. After minimization, MD simulation of 100 ps at 300 K was performed for equilibration and free diffusion of water molecules into the protein structure. Final models were eventually obtained after 2000 steps of minimization (steepest descent). The temperature and pressure of the simulation were controlled by a Nose–Hover chain of thermostats. The cut-off for the computation of nonbonded interactions was set to 12 Å, and the electrostatic forces were softened by defining a relative dielectric constant of 2 for the system.

The docking protocol used was based on a soft-restrained MD approach previously described [50,70]. MD simulations (200 ps) were performed at 300 K to thermally equilibrate the substrate and the protein without restraints applied to the system. Then a distance-dependent constraint whose force constant values ranged from 3 to 9 (kcal/mol)/Å^2^ was applied between the heme iron and the substrate hydroxylation site, and MD simulations were performed at 300 K for 200 ps. Equilibration of the docked ligand in the active site was done by releasing the constraint in a final MD run of 200 ps at 300 K. Final minimization (2000 steps, conjugate gradient) was performed to obtain the CYP2J2-substrate complexes. Pictures of models were obtained using the Pymol software.

## Figures and Tables

**Table 1:** Kinetic constants for the oxidation of ebastine by CYP2J2 and its R117 mutants ^a^.

Kinetic Constants	WT	R117K	R117L	R117E
*K*_M_ (µM)	0.3 ± 0.1	0.4 ± 0.1	1.0 ± 0.2	0.1 ± 0.05
*k*_cat_ (min^−1^)	40 ± 5	102 ± 3	12 ± 2	27 ± 3
*k*_cat_/*K*_M_ (min^−1^ µM^−1^)	133	245	12	193

^a^ Kinetic constants were calculated for the formation of hydroxyebastine upon oxidation of ebastine by microsomes of *Sf21* insect cells expressing recombinant WT CYP2J2 or its R117 mutants (conditions described in Experimental Procedures). Values are means ± SD from three independent experiments.

**Table 2:**
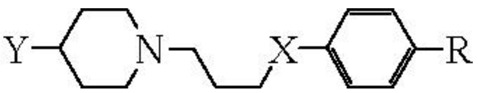
Comparison of the inhibitory effects of terfenadone derivatives towards ebastine hydroxylation by CYP2J2 and its R117X mutants.

Compound	Y-	-X-	-R	IC_50_ (µM) ^a^			
				WT ^b^	R117K	R117L	R117E
			_3_				
Terfenadone **1**	**Ph_2_(OH)C-**	**-CO-**	**--C(CH_3_)_3_**	0.7 ± 0.1	1.6 ± 0.1	1.9 ± 0.2	1.0 ± 0.3
**2**	**Ph_2_(OH)C-**	**-CO-**	**(-(CH_2_)_2_-CH_3_**	0.4 ± 0.1	1.1 ± 0.2	2.4 ± 0.3	0.5 ± 0.3
**3**	**Ph_2_(OH)C-**	**-CH_2_-**	**--C(CH_3_)_3_**	3.6 ± 0.7	8.6 ± 0.2	2.2 ± 0.2	2.1 ± 0.4
**4**	**Ph_2_(OH)C-**	**-CH_2_-**	**--(CH_2_)_2_-CH_3_**	4.5 ± 0.9	13.2 ± 0.1	2.3 ± 0.3	2.9 ± 0.2

^a^ Compound concentration leading to 50% inhibition of ebastine hydroxylation by CYP2J2 and its mutants (see Experimental Procedures; concentrations of ebastine were equal to the *K_M_* values reported in Table 1). Values are means ± SD for three to four independent experiments. ^b^ Values published previously [45].

**Table 3:**
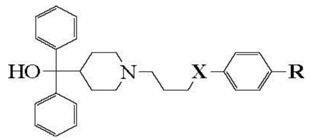
Regioselectivity of the oxidation of the R group of compounds **2**, **4**, and **5** by CYP2J2 and its R117 mutants ^a^.

Substrate			Regioselectivity (%)											
			WT			R117K			R117L			R117E		
	-X-	-R	α	β	γ	α	β	γ	α	β	γ	α	β	γ
**2**	**-CO-**	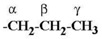	4	**87**	9	4	**92**	4	15	**79**	6	5	**90**	5
**4**	**-CH_2_-**	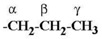	35	**64**	<1	33	**64**	2	44	**52**	4	13	**83**	1
**5**	**-CO-**	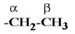	3	**97**		4	**96**		17	**83**		3	**97**	

^a^ Oxidation conditions are described in the Experimental Procedures.

**Table 4:** Distances calculated between the iron and the carbon atoms of the **R** group of compound **2**.

Substrate		*Fe-C Distances (Å)* ^a^											
		WT ^b^			R117K			R117L			R117E		
-X-	-R	α	β	γ	α	β	γ	α	β	γ	α	β	γ
**-CO-**	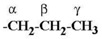	4.9	**3.8**	4.6	5.3	**4.0**	4.4	3.9	**3.6**	3.6	5.4	**4.2**	4.8

^a^ α, β, γ, positions relative to the phenyl ring. ^b^ Values previously reported [50] for wild type CYP2J2.

## Abbreviations

AA	arachidonic acid
CYP	cytochrome P450
EET	*cis*-epoxyeicosatrienoic acid
HPLC	high performance liquid chromatography
MD	molecular dynamics
MS	mass spectrometry
NADP	nicotinamine adenine dinucleotide phosphate
NADPH	reduced nicotinamide adenine dinucleotide phosphate
wt	wild type
